# Evaluation of regional comprehensive development efficiency under low-carbon policy: based on optimized DDF-GML combined with unsupervised clustering method

**DOI:** 10.1038/s41598-024-67236-x

**Published:** 2024-07-13

**Authors:** Runqun Yu, Zhuoyang Luo

**Affiliations:** School of Economics and Management, Dalian Jiaotong University, Dalian, 116028 China

**Keywords:** Green development, Energy efficiency, Environmental benefit, Direction distance function, Environmental social sciences, Energy and society, Sustainability

## Abstract

In the study of urban development, it is very important to evaluate the influence of production factors reasonably and efficiently for the region to achieve efficient development. The principal aim of this investigation is to amalgamate the conventional measurement model characterized by robust interpretability with the non-parametric model characterized by limited interpretability, thereby enhancing the precision of research outcomes. Towards this objective, the study employs an optimized directional distance function integrated with a global Malmquist–Luenberger index to formulate a comprehensive total factor productivity measurement framework. In elucidating the homogeneous attributes of regions, departing from prior methodologies reliant on manual or direct algorithmic partitioning, this paper employs the K-means clustering algorithm for index discernment, abstracting the concept of K-means clustering centroids to encapsulate regional homogeneity, thereby delineating results through the visualization of regional development potential maps and the evolution of centroid-based clustering trend maps. The findings of the investigation illuminate common patterns of change across disparate regions, proposing a strategy for leveraging regional resource endowments towards a cohesive framework, thereby transcending constraints imposed by production efficiency limitations. Amidst the backdrop of the COVID-19 pandemic, this study draws upon provincial-level data spanning from 2000 to 2018 in China. The conclusive analytical outcomes underscore the pivotal role of energy factors in regional development efficiency, particularly within high-potential development regions, followed by the capital and labor factors. Concurrently, the study discerns a discernible hierarchical pattern among areas of development potential, which exhibits correlation with factor mobility dynamics.

## Introduction

In recent years, with the large-scale development and use of primary energy such as coal and oil, the imbalance of regional industrial structure, the excess supply of low-end energy, and the low efficiency of factor use^1^ have become the norm, and the sustainable development of society has been seriously affected. Therefore, people are beginning to realize the importance of green development.

The concept of green development can be traced back to the British environmental economist David Pierce's " Blueprint for a Green Economy " published in 1989. In this book, Pierce first systematically expounded the concept of green economy, marking the beginning of the exploration of the path of green development. At this stage, the discussion of green development mainly focuses on environmental protection, emphasizing the harmonious coexistence between economic activities and ecosystems^2^. Until 2010, the United Nations Environment Programme (UNEP) further expanded green development from the aspects of "human happiness and social equity", and finally formed a balanced framework of environment, economy and society.

**Figure 1:**
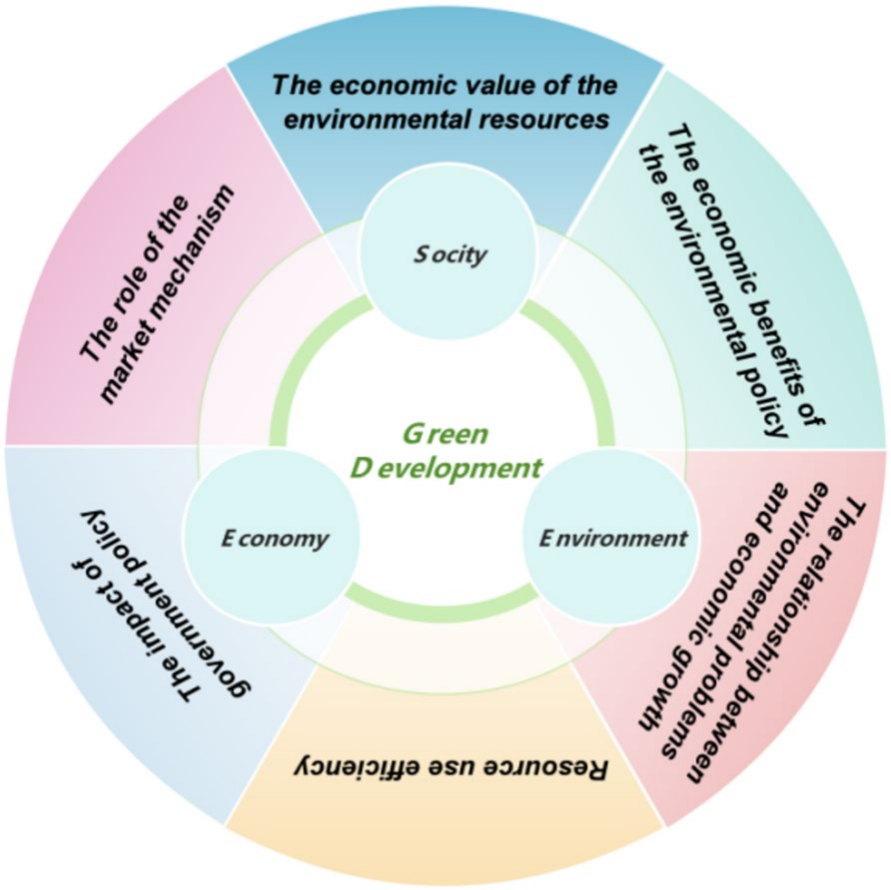
Research perspective of green development.

For the study of green development (see Fig. 1), the academic community currently generally uses a variety of perspectives and methods for comprehensive consideration from the three perspectives of environment, economy and society^3–7^. Among these methods, eco-efficiency is one of the main tools to measure the green development of regions and cities^8^, which is widely used in the calculation of urban green development efficiency and the identification of influencing factors. Under the condition of limited quantity of production factors, technological innovation is often the fundamental way to improve the efficiency of urban green development^9^. By improving the utilization rate of resources, it can effectively alleviate people 's dependence on natural resources and promote the development of green economy^10^. Further, from the perspective of ecological modernization theory, the solution of environmental problems can be achieved through technological innovation-driven environmental regulation^11^, that is, there is a positive impact relationship between technological innovation and environmental regulation. However, in fact, different scholars have different understandings of the relationship between environmental regulation and technological innovation. For example^12^, combined with the DEA-SBM model and the GML index, the relationship between financial development and green technological innovation in 30 provinces of China was investigated. It was found that financial structure played a positive role in green technological innovation, and financial scale and financial efficiency played a negative role in green technological innovation. At the same time, environmental regulation has a positive moderating effect between financial structure and green technology innovation, but it plays a negative moderating role between financial efficiency and green technology innovation^13^ discusses the relationship between environmental regulation and technological innovation from the perspective of China, and finds that the impact of environmental regulation on technological innovation presents a U-shaped relationship, and in the short term, environmental regulation has a ' offset effect ' on the research and innovation capabilities of China 's industrial sector. However, with the strengthening of environmental protection supervision, the industry is forced to reduce costs, so as to control pollution by improving technological innovation ability, which in turn has a ' compensation effect '. It is also found that there is a U-shaped relationship between environmental regulation and technological innovation, and it is proposed that China 's green innovation efficiency presents different regional differences. On the whole, China presents the overall trend of ' rise in the east, stability in the middle and decline in the west '. Environmental regulations exert a significant positive impact on GTFP (green total factor productivity)^14^ find local environmental governance also makes notable contributions to the GTFP of adjacent regions. Furthermore, environmental regulations indirectly promote GTFP by elevating the level of green technological innovation. Results concerning regional heterogeneity indicate that environmental regulations not only directly facilitate GTFP but also indirectly and significantly enhance GTFP in the eastern and central regions through green technological innovation, whereas such effects are not significant in the western region^15^ By analyzing the policy of air pollution reduction, this paper reveals the impact of environmental regulation on technological innovation, and finds that environmental regulation can promote technological innovation, and the three factors of government financing, external governance of capital market and R & D intensity may be three potential economic channels for environmental regulation to promote technological innovation. In a word, no matter what kind of influence, the research methods are often carried out from linear or nonlinear, among which the nonlinear method is not very popular with scholars^7^. Under the background of big data era, is there a recognition method that can combine the traditional measurement model with the big data clustering algorithm (unsupervised), and improve the interpretability and accuracy of the research results without artificially dividing the research scope ? This study will further put forward thinking in this direction.

In the choice of traditional models, considering that the ' Paris Agreement ' has set the macro goal of carbon neutrality in the world, and China has begun to explore low-carbon work as early as 2003.In order to study the impact of environmental regulation, this paper will explore the relationship between low-carbon policy and urban development efficiency from the perspective of China based on the environmental regulation of low-carbon policy. Previous studies on the efficiency of urban development are mainly measured by technological progress. Technological progress was originally proposed by Solow^16^ and gradually evolved into the key idea of total factor productivity measurement. With continuous iteration, the measurement model of total factor productivity has developed from the general data envelopment analysis (DEA)^17^. A directional distance function model that can simultaneously measure the reduction of input and undesired output^18^ provides a research method for measuring pollution emissions. Although the measurement of total factor productivity is the mainstream way to study the efficiency of urban development^19^, the research elements introduced by different research institutes are not the same. For example^18^, capital and labor are used as factor inputs, land, energy and water are used as resource inputs, GDP is used as expected output, and SO2 is used as undesirable output^19^ Labor, capital, energy, resources, etc.are selected as input factors, healthy environment, healthy society, health services, healthy people, healthy culture, etc.are selected as expected outputs, and industrial wastewater discharge and industrial sulfur dioxide are selected to measure undesirable outputs^20^. Taking labor, energy and capital as input factors, per capita GDP as expected output, industrial waste gas, industrial waste water and industrial dust emissions as undesired output^21^ selected capital, labor, energy as input, GDP as expected output, CO2 as undesirable output. It can be found that most of the research is based on the two categories of capital and labor, adding energy or resources as input factors, economic factors or other factors as expected output, and pollution emissions as undesirable output. Since the United Nations Sustainable Development Summit held in Johannesburg, South Africa in 2002, the importance of energy in achieving sustainable development has been widely recognized, and people have gradually realized that it is closely related to carbon emissions^22^. Therefore, based on the input of capital and labor factors, this study incorporates energy factors as input factors, GDP as expected output, and CO2 emissions as undesired output to study the changes in urban comprehensive development efficiency under the background of low-carbon policies, taking into account the serious impact of the new coronavirus epidemic^23^. The data selected in this study are the data of provincial areas in China from 2000 to 2018.

Finally, this study constructs the optimized direction distance function combined with the global Malmquist-Luenberger model. Based on the transformed index, the K-means clustering algorithm is used for further unsupervised identification. Through the clustering of the clustering algorithm, the areas in the study are further divided. In order to effectively identify the impact, the input factors are controlled in the analysis part, and the labor, capital and energy factors are only clustered, that is, the impact of each factor on the change of total factor productivity is identified, and further analysis is proposed according to the identification results. Although the clustering algorithm will weaken the interpretability of the results^24^, its logical premise is based on the results of the traditional model, so this can greatly improve the credibility of the results and break through the situation of artificial division of the recognition range. For example, from the perspective of geographical division, the general research results divide the calculation results into ' western region ', ' central region ', ' eastern region ', ' northwest region ', ' southwest region ', etc.^25,26^ for identification, but through the clustering algorithm, different regions and the same technology development can be gathered together. This provides a reference for studying the linkage effect of inter-regional development or homogeneous factors^27,28^. At the same time, the clustering results can greatly weaken the average effect of factors with large differences among different regions, so that the prominent influence of the identified objects is homogenized, which provides a new perspective for amplifying the role of influencing factors.

**Figure 2:**
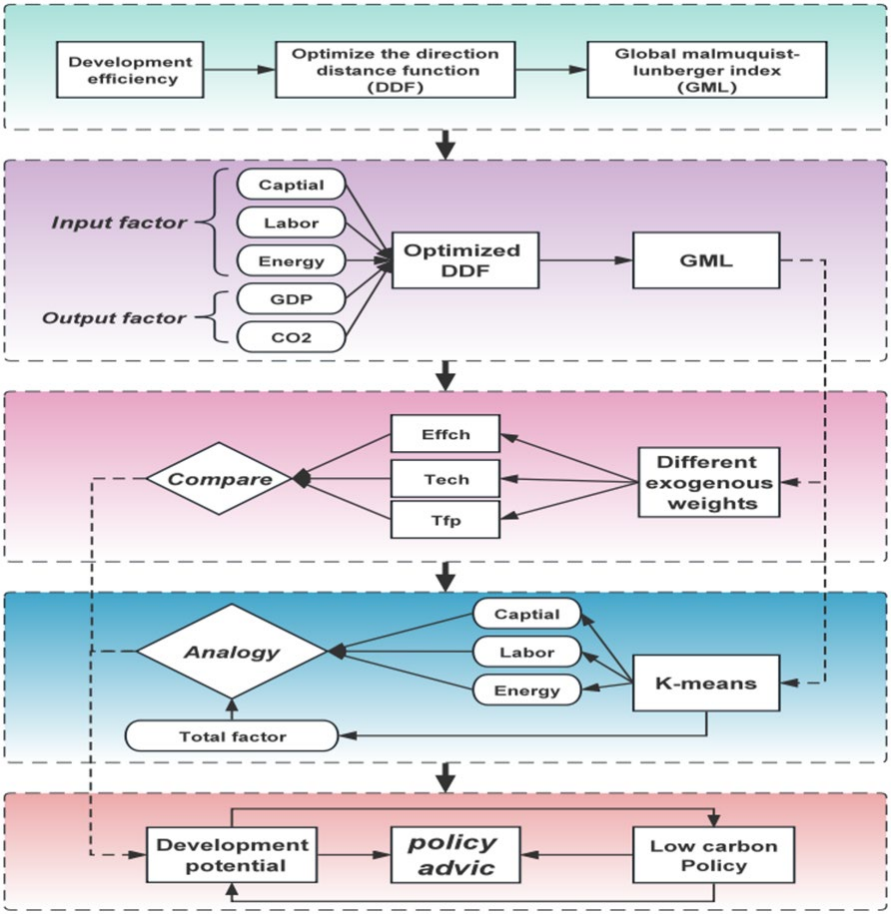
Logic structure diagram.

The main innovations of this study are as follows: (1) Using exogenous weights, by setting different weight combinations to represent different constraint sizes, the importance of each dimension can be intuitively reflected, and the influence of other elements can be measured by setting some elements to 0 weights. (2) By reconstructing the direction vector, the constraint of the undesirable output part is set to an equal form, so that there is no slack variable in the undesirable output part, which can effectively avoid the problem of numerical overestimation. (3) The global Mamquist-Lunberg model is introduced to further accurately measure the impact of energy utilization on the environment and economic benefits through the index model, which provides a solid foundation for guiding the adjustment of energy utilization structure. (4) A new way to realize the homogeneity classification of urban areas is constructed. Using the advantages of K-means clustering method and unsupervised learning, the optimized DDF-GML model is integrated to realize the integration of urban agglomerations in the development direction and the division of transformation potential, which provides a reference for future development (see Fig. 2 for the research framework).

## Methods

### Optimize the DDF model

This study uses the DDF method as a preliminary reference to measure the impact of the energy use structure of each city on the environmental and economic benefits in the context of low-carbon policies. Among them, each city unit is regarded as a production decision-making unit, each decision-making unit has labor input (l), capital input (k) and energy input (e), such as three aspects of input, as well as expected output (y) and undesired output (b). Then, the set of production possibilities can be initially expressed as:

1 $$T = \left\{ {\left( {k,l,e,y,b} \right):\left( {k,l,e} \right){\text{ output}}\left( {y,b} \right)} \right\}$$

According to the existing research of Färe^29^, production technology should meet three basic assumptions, (1) expected output and undesired output have null-jointness; (2) The input factors and expected output have strong disposeability; (3) Undesirable output has weak disposability. Therefore, for the DMU of n decision-making units, the model of the directional distance function is further expressed as:

2 $$\begin{aligned} \mathop D\limits^{ \to } (k,l,e,y,b;g) & = \max \beta \\ & s.t.\;\sum\limits_{n = 1}^{N} {\lambda_{n} k_{n} \le k(1 - \beta )} ,\sum\limits_{n = 1}^{N} {\lambda_{n} l_{n} \le l(1 - \beta )} ,\sum\limits_{n = 1}^{N} {\lambda_{n} e_{n} \le e(1 - \beta )} ,\sum\limits_{n = 1}^{N} {\lambda_{n} y_{n} \ge y(1 - \beta )} ,\sum\limits_{n = 1}^{N} {\lambda_{n} b_{n} = b(1 - \beta )} ,\sum\limits_{n = 1}^{N} {\lambda_{n} } = 1,\lambda_{n} \ge 0,n = 1, \ldots ,N. \\ \end{aligned}$$

Here, $\lambda_{n}$ represents the weight of each decision making unit. $\sum\limits_{n = 1}^{N} {\lambda_{n} } = 1$ represents that the sum of all the weight variables is 1, which can make the production technology become variable returns to scale (VRS). If this restriction is not added, then it means that the return to scale is constant (CRS). Finally, the obtained $\beta$ objective function is the inefficiency value, and $1{ - }\beta$ is the efficiency value.

Although the directional distance function (DDF) has been applied to a certain scale in the process of measuring productivity, its model assumptions still have several drawbacks, that is, limiting the increase of expected output and the reduction of factor input and undesired output is the same proportion. This assumption can easily lead to ' slack bias '. In view of the existing unreasonableness, this study attempts to make further improvements from two aspects: (1) Reconstruct the undesirable output into weak disposability, so that there are no slack variables in the undesirable output part. (2) Introducing exogenous weight $\omega_{i}$ ($\sum {\omega = 1}$). Different weight combinations represent different constraints and objectives, so as to reflect the different importance of each dimension.

Through the improvement of these two aspects, the optimal direction distance function for measuring the efficiency of urban comprehensive transformation is :

3 $$\begin{aligned} \mathop D\limits^{ \to } (k,l,e,y,b;g) & = \max \left( {\omega_{{\text{k}}} \cdot \beta_{k} g_{k} + \omega_{{\text{l}}} \cdot \beta_{l} g_{l} + \omega_{{\text{e}}} \cdot \beta_{e} g_{e} + \omega_{{\text{y}}} \cdot \beta_{y} g_{y} + \omega_{{\text{b}}} \cdot \beta_{b} g_{b} } \right) \\ & {\text{s}}.{\text{t}}.\;\sum\limits_{n = 1}^{N} {\lambda_{n} } k_{n} \le l - \beta_{k} g_{k} ,\sum\limits_{n = 1}^{N} {\lambda_{n} } l_{n} \le k - \beta_{l} g_{l} ,\sum\limits_{n = 1}^{N} {\lambda_{n} } e_{n} \le e - \beta_{e} g_{e} ,\sum\limits_{n = 1}^{N} {\lambda_{n} } y_{n} \ge y + \beta_{y} g_{y} ,\sum\limits_{n = 1}^{N} {\lambda_{n} } b_{n} = b - \beta_{b} g_{b} ,\sum\limits_{n = 1}^{N} {\lambda_{n} } = 1,\lambda_{n} \ge 0,n = 1,...,N \\ \end{aligned}$$

Since the objective function $\beta$ and the direction vector $g = (g_{k} ,g_{l} ,g_{e} ,g_{y} ,g_{b} )$ are variables that require solutions, and the two are in the form of a product, the model (3) is a nonlinear programming problem, which may produce a non-global optimal solution or no solution. According to the conclusions of Färe and Chung^29,30^, let $S_{k} = \beta_{k} g_{k}$, $S_{l} = \beta_{l} g_{l}$, $S_{e} = \beta_{e} g_{e}$, $S_{y} = \beta_{y} g_{y}$, $S_{b} = \beta_{b} g_{b}$, transform the model (3) into a linear programming problem, that is, obtain the formula (4):

4 $$\begin{aligned} \mathop D\limits^{ \to } (k,l,e,y,b;g) & = \max (\omega_{k} \cdot S_{k} + \omega_{l} \cdot S_{l} + \omega_{e} \cdot S_{e} + \omega_{y} \cdot S_{y} + \omega_{b} \cdot S_{b} ) \\ & {\text{s}}.{\text{t}}.\;\sum\limits_{n = 1}^{N} {\lambda_{n} } k_{n} \le l - S_{k} ,\sum\limits_{n = 1}^{N} {\lambda_{n} } l_{n} \le k - S_{l} ,\sum\limits_{n = 1}^{N} {\lambda_{n} } e_{n} \le e - S_{e} ,\sum\limits_{n = 1}^{N} {\lambda_{n} } y_{n} \ge y + S_{y} ,\sum\limits_{n = 1}^{N} {\lambda_{n} } b_{n} = b - S_{b} ,\sum\limits_{n = 1}^{N} {\lambda_{n} } = 1,\lambda_{n} \ge 0,n = 1,...,N\;{\text{and}}\,0 \le S_{k} ,S_{l} ,S_{e} ,S_{y} ,S_{b} . \\ \end{aligned}$$

In summary, based on model (4), this study measures and compares the comprehensive transformation efficiency of Chinese cities, and uses the setting of exogenous weight differentiation to focus on the comprehensive benefits of energy use and environmental protection in the target areas.

### The construction of global Malmquist–Luenberger exponential function

Färe (1989) defined the productivity index based on Shepard 's output distance function, and Chung adjusted it to obtain the Malmquist–Luenberger index, oh^31^ defined the global reference productivity index with reference to Chung 's method. This study is based on the DDF function optimized in the previous part, combined with the global reference Malmquist–Luenberger index to conduct in-depth comprehensive measurement of urban energy use and environmental protection benefits.

Generally speaking, the form of Malmquist–Luenberger index is :

5 $$\begin{gathered} ML^{t,t + 1} (x^{t} ,y^{t} ,b^{t} ,x^{t + 1} ,y^{t + 1} ,b^{t + 1} ) \hfill \\ = \left[ {\frac{{1 + D^{t} (x^{t} ,y^{t} ,b^{t} )}}{{1 + D^{t} (x^{t + 1} ,y^{t + 1} ,b^{t + 1} )}} \times \frac{{1 + D^{t + 1} (x^{t} ,y^{t} ,b^{t} )}}{{1 + D^{t + 1} (x^{t + 1} ,y^{t + 1} ,b^{t + 1} )}}} \right]^{1/2} \hfill \\ = \frac{{1 + D^{t} (x^{t} ,y^{t} ,b^{t} )}}{{1 + D^{t + 1} (x^{t + 1} ,y^{t + 1} ,b^{t + 1} )}} \times \left[ {\frac{{1 + D^{t + 1} (x^{t} ,y^{t} ,b^{t} )}}{{1 + D^{t} (x^{t} ,y^{t} ,b^{t} )}} \times \frac{{1 + D^{t + 1} (x^{t + 1} ,y^{t + 1} ,b^{t + 1} )}}{{1 + D^{t} (x^{t + 1} ,y^{t + 1} ,b^{t + 1} )}}} \right]^{1/2} \hfill \\ = \frac{{TE^{t + 1} }}{{TE^{t} }} \times \left[ {TG_{t}^{t,t + 1} \cdot TG_{t + 1}^{t,t + 1} } \right]^{1/2} \hfill \\ = EC^{t,t + 1} \cdot TC^{t,t + 1} \hfill \\ \end{gathered}$$

TE measures the technical efficiency, and TG measures the technical gap from period t to period t + 1. EC measures the change of technical efficiency from t to t + 1.

Due to the use of adjacent parameters to calculate the inter-temporal efficiency, there will be super-efficiency, which is prone to the situation of no feasible solution. Therefore, based on the above formula, this study uses the Malmquist–Luenberger exponential function of the global reference, and the calculation formula obtained is as follows :

6 $$\begin{gathered} GML_{t}^{t + 1} = \frac{{1 + \mathop {D_{V}^{G} }\limits^{ \to } (k^{t} ,l^{t} ,e^{t} ,y^{t} ,b^{t} ;g)}}{{1 + \mathop {D_{V}^{G} }\limits^{ \to } (k^{t + 1} ,l^{t + 1} ,e^{t + 1} ,y^{t + 1} ,b^{t + 1} ;g)}} \hfill \\ = \frac{{1 + \mathop {D_{V}^{t} }\limits^{ \to } (k^{t} ,l^{t} ,e^{t} ,y^{t} ,b^{t} ;g)}}{{1 + \mathop {D_{V}^{t + 1} }\limits^{ \to } (k^{t + 1} ,l^{t + 1} ,e^{t + 1} ,y^{t + 1} ,b^{t + 1} ;g)}} \hfill \\ \times \left[ {\frac{{(1 + \mathop {D_{o}^{G} }\limits^{ \to } (k^{t} ,l^{t} ,e^{t} ,y^{t} ,b^{t} ;g)) \, { (1 + }\mathop {D^{t + 1} }\limits^{ \to } (k^{t + 1} ,l^{t + 1} ,e^{t + 1} ,y^{t + 1} ,b^{t + 1} ;g))}}{{(1 + \mathop {D_{o}^{G} }\limits^{ \to } (k^{t + 1} ,l^{t + 1} ,e^{t + 1} ,y^{t + 1} ,b^{t + 1} ;g)) \, { (1 + }\mathop {D^{t} }\limits^{ \to } (k^{t} ,l^{t} ,e^{t} ,y^{t} ,b^{t} ;g))}}} \right] \hfill \\ \end{gathered}$$

Among them, the newly constructed productivity index function can be decomposed into:

7 $$GML_{t}^{t + 1} = \frac{{TE^{t + 1} }}{{TE^{t} }} \times \frac{{BPG_{t + 1}^{t,t + 1} }}{{BPG_{t}^{t,t + 1} }} = EC^{t,t + 1} \times BPC^{t,t + 1}$$

The meaning of TE and EC is the same as the above introduction. BPC measures the gap between the current technology frontier and the global technology frontier.

### Ethical approval

Written informed consent for publication of this paper was obtained from all authors.

## Data

The original index data come from ' China City Statistical Yearbook ', ' China Energy Statistical Yearbook ' and so on.

Among them, the objects of this study are mainly provincial-level regions, including some municipalities directly under the central government. Because the data of some provinces and cities are not complete, the provincial-level regions that have been collated and used are Beijing, Tianjin, Hebei, Shanxi, Inner Mongolia, Liaoning, Jilin, Heilongjiang, Shanghai, Jiangsu, Zhejiang, Anhui, Fujian, Jiangxi, Shandong, Henan, Hubei, Hunan, Guangdong, Guangxi, Chongqing, Sichuan, Guizhou, Yunnan, Shaanxi, Gansu, Qinghai and Xinjiang.

The detailed variable selection and data processing are as follows:

Labor input: The unit 's labor input data selects the natural logarithm of the number of employees as a measure of labor input.

Capital investment: This study selects fixed asset investment as capital investment, and uses the perpetual inventory method to estimate the actual capital stock. The formula is :

8 $$K_{it} = (1 - \sigma_{it} )K_{i,t - 1} + E_{it}$$

Among them, $K_{it}$ represents the capital stock of i-units in t period, $K_{i,t - 1}$ represents the capital stock of i-units in t-1 period, $E_{it}$ represents the fixed asset investment of i-units in t period after index reduction with fixed asset price, $\sigma_{it}$ represents the depreciation rate of fixed assets of i-units in t period. This study refers to Young 's^32^ estimation method of capital stock in base period. Regarding the inter-provincial capital stock in 2000, this study uses the sum of the average depreciation rate of 10.96% in the actual capital formation ratio of each province in 2001 and the average growth rate of investment from 2001 to 2005.

Energy input: In the existing research, most scholars choose the use of electricity as an indicator of energy input (Wei and Song^33^). On the one hand, because the GDP elasticity of electricity demand is very close to the GDP elasticity of energy demand, and electricity has become one of the main forms of energy consumption in China (Lin and Peng, 2017)^34^; on the other hand, the use of other energy sources in China is easily underestimated. The power consumption data directly read by the device is more accurate. Therefore, this study chooses regional electricity consumption as a measure of energy input.

Output variables: measured by regional GDP, through the measurement of GDP to determine whether the region can achieve energy efficiency, environmental protection benefits and economic benefits in the context of the implementation of low-carbon policies.

Carbon dioxide emissions: this study uses the total amount of carbon dioxide emissions in each province as an indicator to measure the benefits of low-carbon policies. According to the 2006 Intergovernmental Panel on Climate Change (IPCC) for the United Nations Framework Convention on Climate Change and Kyoto Protocol developed by the National Greenhouse Gas Inventories Guide Volume II (Energy) Chapter VI of the reference method, the total amount of carbon dioxide emissions can be estimated based on the amount of carbon dioxide emissions caused by various energy consumption. The specific formula is as follows:

9 $$CO_{2} = \sum\limits_{i = 1}^{3} {CO_{2,i} = \sum\limits_{i = 1}^{3} {E_{i} \times NCV_{i} \times CEF_{i} } }$$

Among them, CO2 represents the estimated carbon dioxide emissions, i represents a variety of energy (coal, coke, coke oven gas, blast furnace gas, converter gas, other gas, crude oil, gasoline, kerosene, diesel, fuel oil, liquefied petroleum gas, natural gas and liquefied natural gas), E represents the consumption of energy. NCV comes from the average low calorific value of China 's primary energy in the 2007 ' China Energy Statistical Yearbook ' (In which the average low calorific value of raw coal is selected; other gas according to the average value of other gas listed in the coefficient table). CEF represents the carbon dioxide emission factors of various energy sources, which data is from IPCC (2006), in which the average value of anthracite, coking coal, other bituminous coal, sub-bituminous coal and lignite is taken as the coal; blast furnace gas takes blast furnace gas value; the converter gas takes oxygen to blow the furnace gas value; other gas takes the same value as coke oven gas.

## Results

In this study, the input–output factors are expressed by using different weights. The weight $\left( {{1 \mathord{\left/ {\vphantom {1 {9,{1 \mathord{\left/ {\vphantom {1 {9,{1 \mathord{\left/ {\vphantom {1 {9,{1 \mathord{\left/ {\vphantom {1 {3,{1 \mathord{\left/ {\vphantom {1 3}} \right. \kern-0pt} 3}}}} \right. \kern-0pt} {3,{1 \mathord{\left/ {\vphantom {1 3}} \right. \kern-0pt} 3}}}}}} \right. \kern-0pt} {9,{1 \mathord{\left/ {\vphantom {1 {3,{1 \mathord{\left/ {\vphantom {1 3}} \right. \kern-0pt} 3}}}} \right. \kern-0pt} {3,{1 \mathord{\left/ {\vphantom {1 3}} \right. \kern-0pt} 3}}}}}}}} \right. \kern-0pt} {9,{1 \mathord{\left/ {\vphantom {1 {9,{1 \mathord{\left/ {\vphantom {1 {3,{1 \mathord{\left/ {\vphantom {1 3}} \right. \kern-0pt} 3}}}} \right. \kern-0pt} {3,{1 \mathord{\left/ {\vphantom {1 3}} \right. \kern-0pt} 3}}}}}} \right. \kern-0pt} {9,{1 \mathord{\left/ {\vphantom {1 {3,{1 \mathord{\left/ {\vphantom {1 3}} \right. \kern-0pt} 3}}}} \right. \kern-0pt} {3,{1 \mathord{\left/ {\vphantom {1 3}} \right. \kern-0pt} 3}}}}}}}}}} \right. \kern-0pt} {9,{1 \mathord{\left/ {\vphantom {1 {9,{1 \mathord{\left/ {\vphantom {1 {9,{1 \mathord{\left/ {\vphantom {1 {3,{1 \mathord{\left/ {\vphantom {1 3}} \right. \kern-0pt} 3}}}} \right. \kern-0pt} {3,{1 \mathord{\left/ {\vphantom {1 3}} \right. \kern-0pt} 3}}}}}} \right. \kern-0pt} {9,{1 \mathord{\left/ {\vphantom {1 {3,{1 \mathord{\left/ {\vphantom {1 3}} \right. \kern-0pt} 3}}}} \right. \kern-0pt} {3,{1 \mathord{\left/ {\vphantom {1 3}} \right. \kern-0pt} 3}}}}}}}} \right. \kern-0pt} {9,{1 \mathord{\left/ {\vphantom {1 {9,{1 \mathord{\left/ {\vphantom {1 {3,{1 \mathord{\left/ {\vphantom {1 3}} \right. \kern-0pt} 3}}}} \right. \kern-0pt} {3,{1 \mathord{\left/ {\vphantom {1 3}} \right. \kern-0pt} 3}}}}}} \right. \kern-0pt} {9,{1 \mathord{\left/ {\vphantom {1 {3,{1 \mathord{\left/ {\vphantom {1 3}} \right. \kern-0pt} 3}}}} \right. \kern-0pt} {3,{1 \mathord{\left/ {\vphantom {1 3}} \right. \kern-0pt} 3}}}}}}}}}} \right)$ is used to calculate the changes of comprehensive development technical efficiency and total factor productivity in provincial-level regions under the same importance of energy, labor and capital input. The weight $\left( {{0},{0},{{1} \mathord{\left/ {\vphantom {{1} {{3},{{1} \mathord{\left/ {\vphantom {{1} {{3},{{1} \mathord{\left/ {\vphantom {{1} {3}}} \right. \kern-0pt} {3}}}}} \right. \kern-0pt} {{3},{{1} \mathord{\left/ {\vphantom {{1} {3}}} \right. \kern-0pt} {3}}}}}}} \right. \kern-0pt} {{3},{{1} \mathord{\left/ {\vphantom {{1} {{3},{{1} \mathord{\left/ {\vphantom {{1} {3}}} \right. \kern-0pt} {3}}}}} \right. \kern-0pt} {{3},{{1} \mathord{\left/ {\vphantom {{1} {3}}} \right. \kern-0pt} {3}}}}}}} \right)$ is used to calculate the changes of comprehensive development technical efficiency and total factor productivity in provincial-level regions only considering energy input (Only considering the influence of capital or labor also can be obtained). Finally, based on the data of 2000–2018 in China 's provincial-level regions, under the background of low-carbon development, this study only considers the regional development efficiency of energy impact from the perspective of regional comprehensive development efficiency. And through the clustering algorithm to stratify the four different development validity of the regional group, and use the results of the calculation respectively from the macro policy impact, expected output and undesirable output expansion potential for in-depth discussion (All map pictures are produced by Microsoft office excel 2007).

### Efficiency changes from the perspective of different input factors

**Figure 3:**
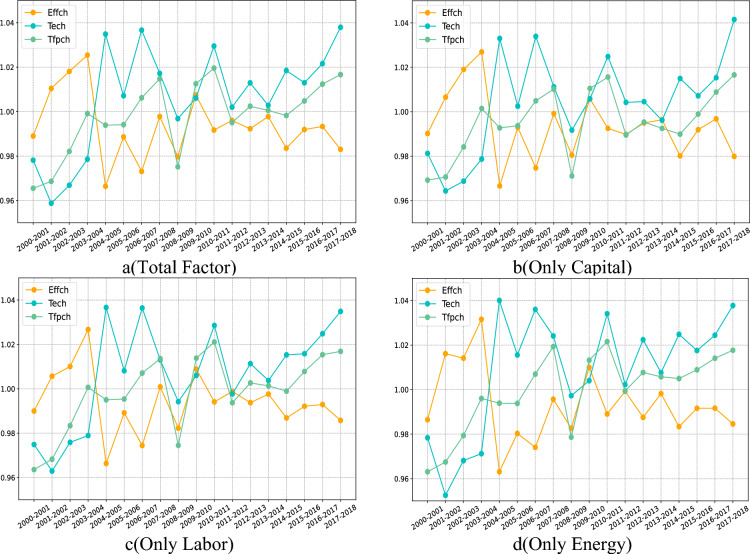
Effects of different factor inputs on changes in total factor productivity.

Figure 3a shows the combined effects of three factors (capital, labor, energy) on technological progress (Tech), technical efficiency (Effch) and total factor productivity change (Tfpch). Figure 3b–d shows that only three factors are considered to affect Tech, Effch and Tfpch separately.

It can be observed that whether considering individual factors or total factor inputs, the three indicators show a similar trend of change. The only difference between them is the magnitude of change at each turning point. For example, when considering the total factor and the three factor inputs separately, the measurement results of total factor productivity changes are almost similar. However, when only energy factors (electric energy) are considered, the trend of change is relatively slow, indicating that energy factors have little impact on total factor productivity. When considering other factors, the change is larger, and the influence of capital factor is relatively more significant. In terms of technological progress, the situation between 2002 and 2003 shows that the changes in technological progress are basically similar when considering total factor input and only capital input. Similarly, only considering labor or energy, the changes in technological progress are similar. However, when considering total factors and only considering capital investment, the change growth is relatively slow, while when only considering labor or energy, the technological progress shows a sudden growth. Subsequent changes also show a trend of sudden growth, which shows that capital factors have a greater impact on total factor productivity in the early stage, while in the later stage, it is more comprehensive. The technical efficiency part shows a similar trend to technological progress, but the overall trend of volatility reduction is finally presented, especially when only capital investment is considered.

In the two time periods from 2004 to 2005 and from 2008 to 2009, regardless of the input of any factor, technological progress and technical efficiency have shown dramatic mutations. Especially when only capital or labor is considered, the change is more intense than when only energy and total factors are considered. This shows that in these two time periods, there are wider and more influential factors, and have a significant impact on total factor productivity, especially for capital and labor factors.

### Visualization of different development potential areas

By clustering the total factor productivity of each provincial region, this part constructs four parts of development potential (high potential, higher potential, general potential, low potential). It should be noted that the index of this clustering is the total factor productivity index. When the value is greater than 1, the total factor productivity growth is considered, and when it is less than 1, it is reduced^35^. This paper holds that the change of total factor productivity is divided into internal influence and external influence. Previous studies mainly focus on identifying the change of total factor productivity and its influencing factors. In this study, through clustering and visualization, the commonness of the change of total factor productivity in each provincial-level region is aggregated by means of relative comparison, that is, the change of total factor productivity in the same color region is similar. Then, under the condition of controlling the respective influence and overall influence of the three input factors, we can find the potential interaction between the advantageous region and the disadvantageous region on the basis of the original research to identify the change. And the potential collaboration of regions with different factor advantages may be possible (so even if the total factor productivity change of all regions is less than 1, if the relative advantage is discovered, it provides the possibility of advantage synergy, for example, in the direction of capital, some regions are high potential regions, while in the direction of labor, they are low potential regions, so other regions with the opposite situation can build cooperative relations with these regions and improve the overall total factor productivity), which will provide visual guidance for the high-quality development of the region.

**Figure 4:**
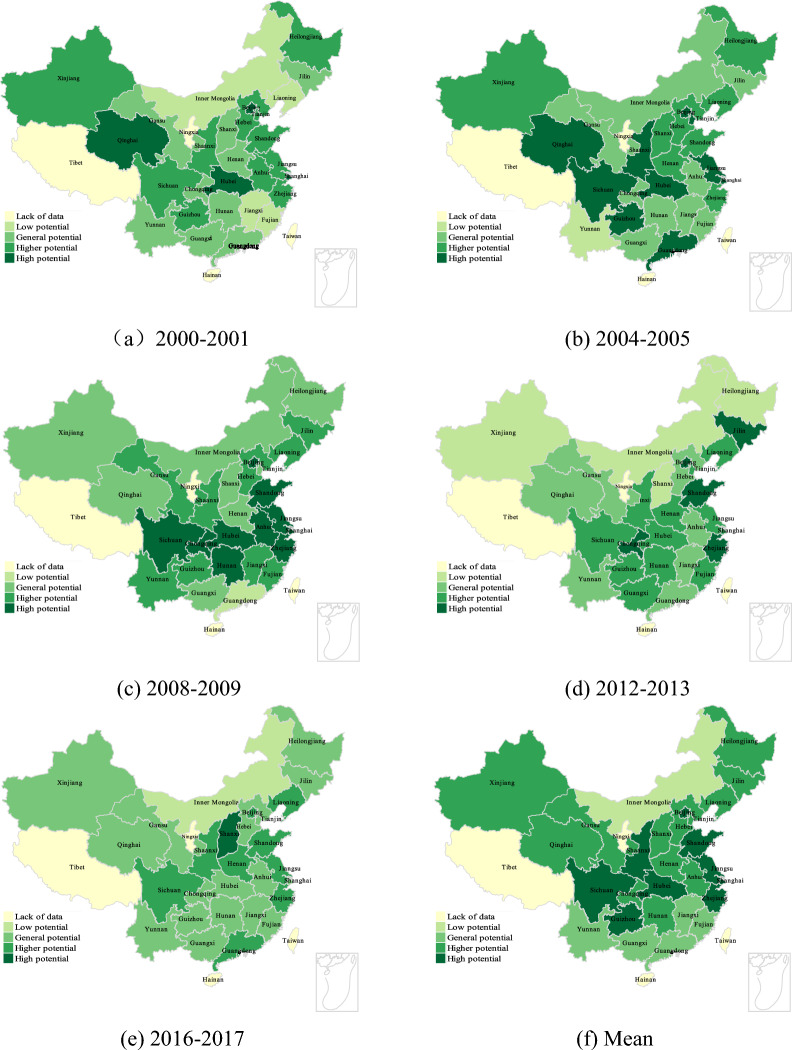
Clustering of development potential of provinces considering only the influence of capital factors.

Figure 4a–f shows the development potential evaluation of five time nodes with uniform interval in China 's provincial regions under the premise of only considering the input of fixed asset investment, and the development potential area display after the average division. It can be found that although the distribution of total factor productivity changes over the years is not uniform, there is a certain rule, that is, the potential distribution of each provincial region basically presents a ladder distribution from south to north, among which the development potential of southern and northern China is weak, and the development potential of central China is large. Therefore, in the case of only considering the capital factor, the areas with great advantages in development potential are mainly distributed in central China, and its core is mainly concentrated in Shaanxi, Sichuan and Hubei. It shows that capital investment has a greater impact on the total factor productivity of these areas and their surrounding areas, while the impact on Northeast China (Heilongjiang, Jilin, Liaoning, etc.), Western and Northwest China (Xinjiang, Gansu, Qinghai, etc.) is relatively weak, and the impact on economically developed coastal areas is the weakest. Among them, the Inner Mongolia region is in a low development potential situation for most of the time, which may be due to its region, land, infrastructure and other reasons, resulting in the weakening of development potential^36^. Since this part only focuses on the impact of capital factor input, it is impossible to discuss the comprehensive situation of various factors horizontally. In the next few parts, we will combine the analysis of the previous part to discuss the comprehensive effect of total factor productivity.

**Figure 5:**
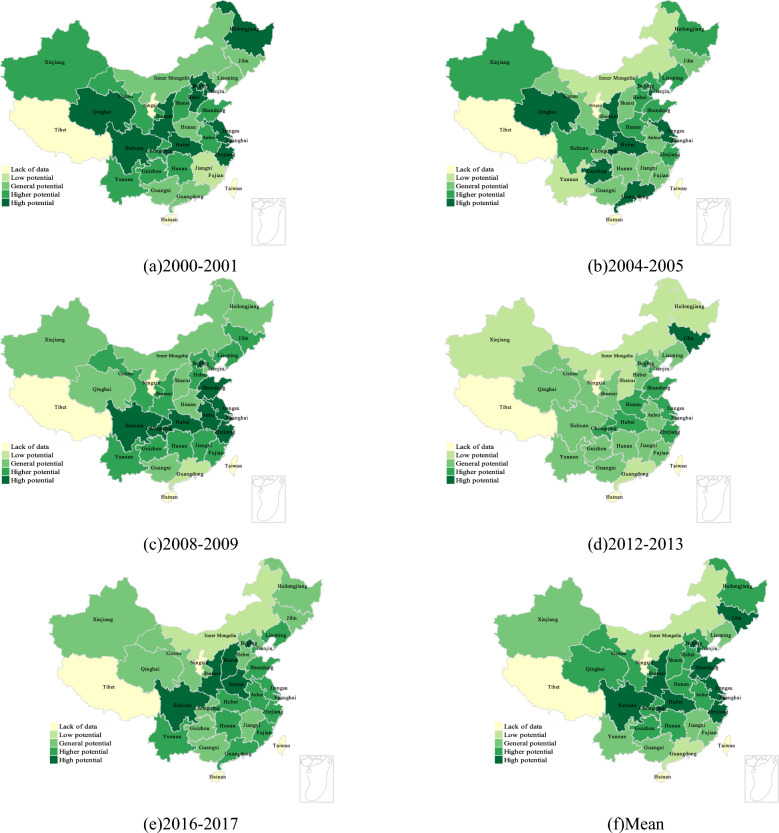
Clustering of development potential of provinces considering only the influence of labor factors.

Figure 5a–f shows the development potential evaluation of five time nodes with uniform interval in China 's provincial regions under the premise of only considering the input of number of employees, and the display of development potential areas after the average division. First of all, an interesting phenomenon is that in the case of only considering the impact of labor factors on total factor productivity, the final distribution of provincial-level regions is partially similar to the distribution in Fig. 4, but more of a difference. It can be found that in the case of only considering the influence of labor factors, the change of total productivity is also similar to the ladder distribution, but the change of potential regional distribution (from high potential to low potential) changes from a south-to-north trend depicted in Fig. 4 to a north-to-south trend, especially in western and northwest China (Xinjiang, Qinghai, etc.) and northeast China (Heilongjiang, Jilin, Liaoning, etc.), which is in line with the situation of China 's population transfer^37,38^. It further shows that in the relative case, through the way of capital investment, it is reasonable to first drive the development of human resources loss areas such as northeast and northwest. Further analysis of the visualization results shows that the south-central region of China seems to be a region where labor factors play a key role in the change of total factor productivity. Except from 2012 to 2013, the development potential of Shaanxi, Hubei and Sichuan provinces is at a high level in most of the time. This shows that labor factors play an important role in the development of total factor productivity in these regions. Similarly, in southern China, especially in Guangdong, except for the two periods of 2004–2005 and 2016–2017, the impact of labor factors is more significant, and the rest of the time shows a lower efficiency state. This is very similar to the results of only considering the impact of capital factors on total factor productivity, reflecting the correlation between capital factors and labor factors^39,40^, and suggesting that factor allocation may have a certain impact on total factor productivity changes.

**Figure 6:**
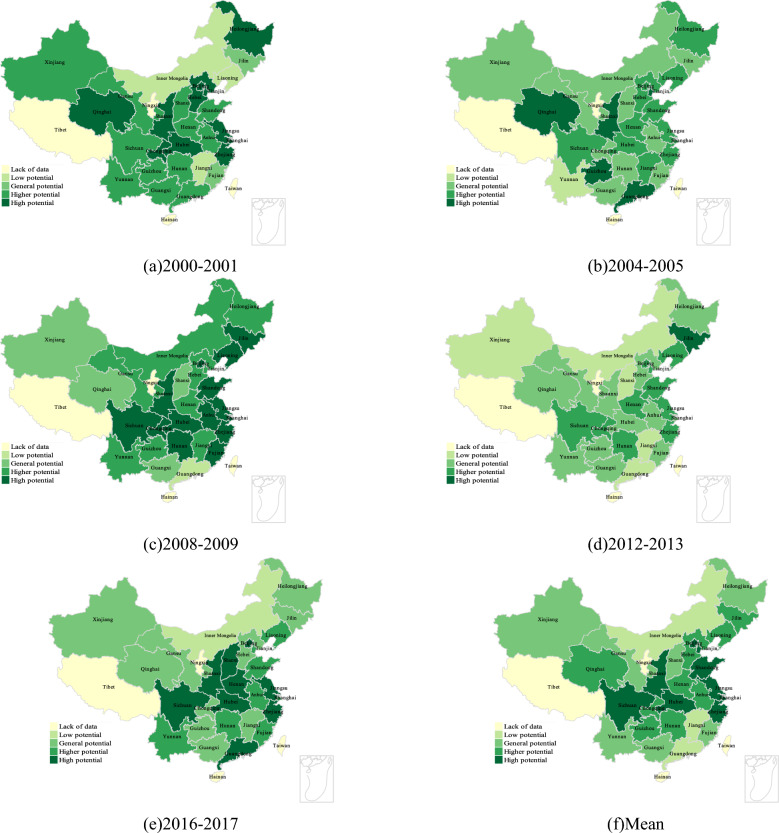
Clustering of provincial development potential considering only the influence of energy factors.

Figure 6a–f shows the development potential distribution of each provincial region under the influence of energy (electricity) factors only. It can be found that under the influence of energy only, the change of regional development potential is basically similar to that of labor and capital, but the difference is that the change of regional potential shows two blocks, one is the high potential development area around the western region (Sichuan, Shaanxi, etc.), and the other is the coastal economic belt (Guangdong, Jiangsu, Zhejiang, etc.). Since the energy input introduced in this study is the power factor, it reflects the distribution of China 's west–east power transmission project to a certain extent, and reflects the credibility of the research results from the side. Through analysis, it can be found that the potential development areas are mainly coastal areas (Guangdong, Zhejiang, Jiangsu, etc.) and the central and western regions (Shaanxi, Sichuan, Hubei, etc.). For coastal areas, coastal areas usually have more industries and manufacturing industries, and these industries have greater demand for electricity. Therefore, the stability and adequacy of power supply are crucial to its economic development, so it may make the regional development potential more dependent on electric energy^41^.For the central and western regions, as their resources are abundant and the output of thermal power capacity is the eastern region. The content of this calculation includes some energy sources of carbon emissions (non-renewable energy sources such as coal and coke), so for the high potential development of the western region, the possible influencing factors are the transformation of energy use structure under low-carbon policies, industrial restructuring, infrastructure construction, etc.^41–43^.

**Figure 7:**
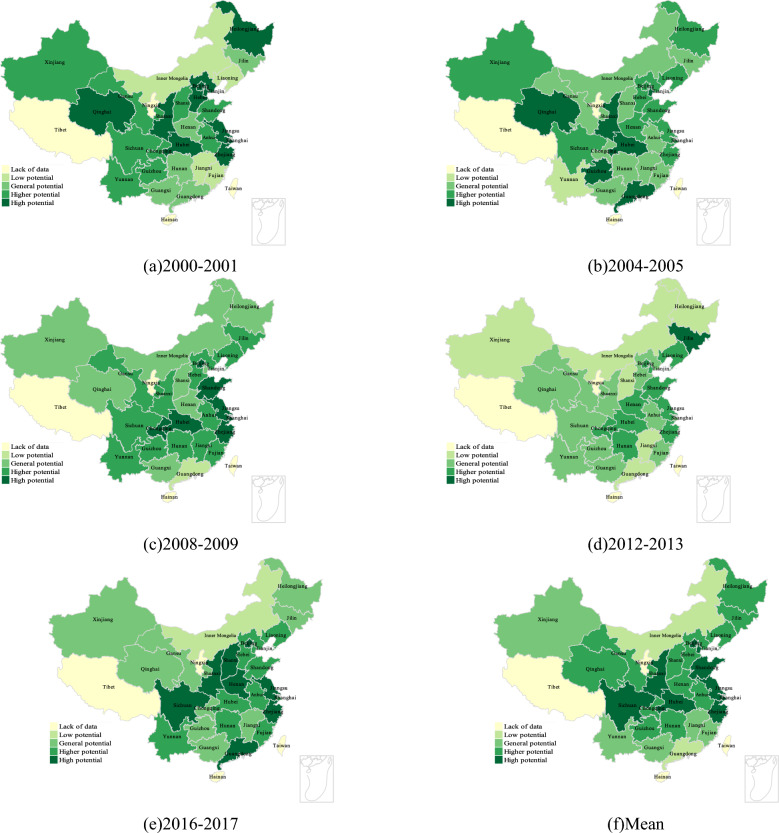
Clustering of development potential of provinces considering only the influence of total factors.

Figure 7a–f shows the development potential distribution of each provincial region under the influence of total factors (capital, labor, energy). It can be found that whether it is analyzed from the time distribution (2000–2017) or the mean distribution, there will be high potential development in the central and western regions of China, especially in Shaanxi, Sichuan and Hubei provinces. Around these three central regions, China shows a trend of potential change from north to south and from east to west (from high potential to low potential), which further reflects the guiding role of economically developed regions in development. Combined with the analysis of the first three parts, it can be found that from the perspective of total factor productivity, the influence of the three input factors is controlled, and the general distribution is the same. When there are special differences in each part, from the perspective of the total factor productivity measurement framework, the potential impact of policies and systems can be obtained. For example, the situation of the high-potential distribution area in the energy part is similar to the distribution area of China 's West–East power transmission project, and the distribution of the impact of electric energy on economic development in the eastern region and the central and western regions summarized in previous studies is the same^43^; In the labor part, the distribution of regional development potential is similar to the trend of China 's population migration^37^. At the same time, it may also be due to the solid foundation of heavy industry in the northeast and northwest regions, and the extensive use of primary energy. The impact of low-carbon policies has also begun to appear related^38^. Considering the lack of quantitative analysis for the interpretation of policies and systems, and the lack of accurate quantitative standards, from the perspective of the correlation of big data rather than the principle of causality, if the introduction time of low-carbon policies is continuous with the change time of the influencing factors of low-carbon policies in most parts of China, it is likely to be the impact of low-carbon policies (if the amount of data tends to infinity, and the differences in regional characteristics tend to infinity, then it will be effectively proved). Therefore, in the next part of this paper, further analysis will be carried out by means of time series broken lines.

### The change of total factor productivity index in different development potential areas

In this part, through the cluster analysis of the total factor productivity (TFP) change value of each development potential area, the purpose is to reveal the characteristics of the center value change in different potential areas. Specifically, we use the clustering algorithm to systematically classify the TFP data of each region, so as to identify the change pattern of the representative center value (cluster center). By analyzing the changes of these center values, we can deeply understand the differences in efficiency and innovation ability of different regions in the process of economic development. This process not only reveals the dynamic changes of total factor productivity in each region, but also provides an important basis for the formulation of regional economic policies, and plays a supplementary role in the visual analysis of the previous part.

**Figure 8:**
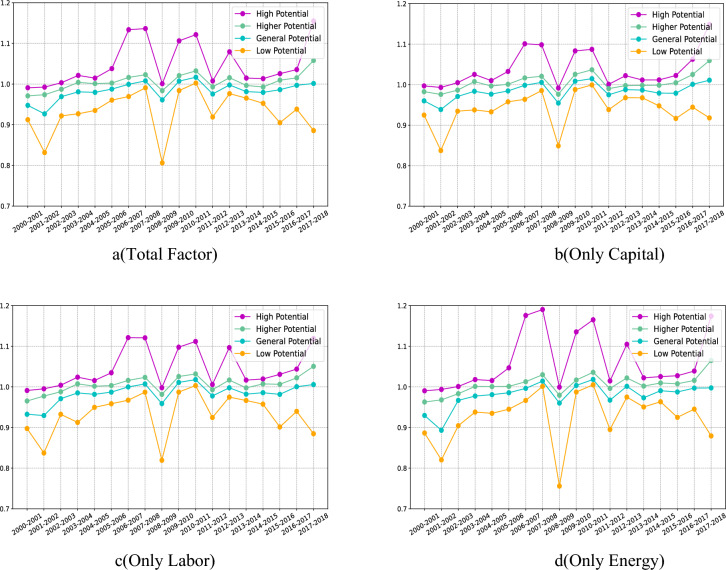
Changes in the cluster center value of each development potential area.

Figure 8a–d shows the impact of considering the comprehensive input of factors (capital, labor, energy) and considering the impact of three kinds of factor inputs (capital, labor, energy) on the change of total factor productivity. It can be found that no matter which part of the results, there were dramatic changes in 2001–2002 and 2008–2009, indicating that a certain factor affected all the elements, and this effect was particularly obvious for high development potential and low development potential areas, such as the introduction of low-carbon policies, the world economic crisis and other emergencies, but no matter what kind of potential factors, it does not affect the analysis of the role of input factors. At the same time, according to the image content, it can be found that for low development potential areas, no matter what kind of factor input, its role is gradually weakening. According to the content of the visual map part, it can be found that some of the low potential areas (Inner Mongolia, Heilongjiang, etc.) belong to the backward impact of infrastructure, and the other part belongs to the development saturation area (Guangdong, etc.), so the impact of factor input is relatively weak. The changes in total factor productivity in areas with general development potential and high development potential are relatively stable. Combined with the previous part, it can be found that these areas are mainly located in central China (Chongqing, Hubei, Henan, etc.) and Liaoning coastal areas, which belong to the transition zone from high development potential areas to low development potential areas. The tolerance of emergencies is relatively high, but it also reflects the impact of some sudden changes.

Overall, energy (electricity) has a relatively low impact on the improvement of total factor productivity in low-potential areas, and has a greater impact on areas with higher potential and above, especially in high-potential areas. The impact of capital and labor on low-potential areas is slightly greater than that of energy factors, among which capital factors play a greater role. Therefore, for low potential and general potential areas, in order to improve the overall development level, we can give priority to the use of capital factors, followed by labor factors, and finally energy factors. For high potential and high potential areas, we should first optimize the use of energy factors, followed by labor factors, and finally capital factors.

## Conclusion

In the context of low-carbon policies, fully assessing the comprehensive development potential of the region can effectively help the region achieve comprehensive development. Through the investigation of previous research, it is found that most of the research is mainly carried out from three perspectives of society, economy and environment. The core idea is to explore the role of technological innovation and policy and their synergistic effects. In previous studies, the measurement of policy impact is mainly the difference-in-difference method^44^, and the evaluation of urban efficiency is mostly data envelopment analysis^45^. Because machine learning algorithms can dynamically evaluate the impact of different factors on the comprehensive development potential of the study area, many studies have begun to explore the integration model of machine learning algorithms^46^. Different from previous studies, this study believes that the policy is a re-adjustment of the regional development situation around the actual situation. At the same time, because the policy has a wide range of influence, its real situation cannot be accurately measured. Therefore, this study aims to use the principle of big data algorithm ' correlation rather than causality ' to explore a research on the relationship between regional development potential and total factor productivity, as well as the correlation between policies.

First of all, in order to avoid the problem of poor interpretability of the big data model^47^, based on the optimization of exogenous weight and direction vector, this study improves the direction distance function, and uses the global Malmquist–Luenberger index to evaluate the total factor productivity, and preliminarily processes the indicators through traditional methods. On this basis, the K-means clustering algorithm is introduced to divide all kinds of development potential areas through the indicators independently constructed by the computer, rather than through the previous manual division. This greatly improves the accuracy of the division of homogeneous feature areas, and largely eliminates the interference of the average situation. Inspired by the clustering algorithm, this study uses the visual map to show the spatial and regional characteristics of different development potential areas, and at the same time refers to the K-means clustering to construct data clusters by exploring the centroid. The centroid (clustering center) of the total factor productivity index in different regions in the K-means clustering process is extracted. This study believes that the clustering center can reflect the common change characteristics of the same potential area to a great extent in theory. Therefore, the centroid values of different potential areas are further used to construct a line chart, which clearly reflects the influence of different factors and the reflection (mutation) of the macro effect in the low dimension. We believe that the greatest contribution of this study is to return the method to its mathematical structure. In the context of the era of big data, it provides useful thinking for the future in-depth construction of research that conforms to the characteristics of ' data '.

Based on the analysis results, this study found four phenomena:

1. The influence of energy factor (electric energy) is relatively stronger. Whether it is the distribution of the development potential of each region or the change of the central characteristics of each development potential region, it shows that the energy factor has a stronger influence than the capital and labor factors. Especially for high potential development areas, followed by high potential development areas, the impact is relatively weak for general development potential areas, while for low development potential areas, the impact of energy factors is significantly lower than that of labor and capital factors. In terms of geographical distribution, the regional distribution that is greatly affected by energy factors is mainly in the western and eastern regions of China, that is, resource-rich and economically developed regions, reflecting the influence of cross-regional resource endowments.
2. Capital factors play a key role in regional development. In general, the capital factor has a relatively large impact on the development of all types of development potential areas, among which the high development potential areas are more important, followed by the high development potential areas, and the impact on other development potential areas is relatively general. In terms of geographical distribution, the regional distribution that is greatly affected by capital factors presents a stepped shape from south to north, which is in line with the staggered transition of China 's developed regional economy to economically underdeveloped regions.
3. The impact of labor factor is greater than that of capital factor in the medium-term stage, and the current impact is less than that of capital factor. This further illustrates the trend of saturation of China 's working population and its requirements for the deep adjustment of productivity structure. From its map visualization development trend, it can be found that its changes are in line with the direction of China 's population transfer (population migration from northeast China, population aggregation in coastal areas of Guangdong and Zhejiang).
4. The change of the total factor productivity index is basically close to the distribution of the influence of energy factors, indicating that the influence of energy factors is greater than that of other factors. At the same time, it is interesting that only considering the influence of capital factors, the regional development potential basically shows a ladder from south to north, and only considering the influence of labor factors, it shows a vague trend from north to south, and the high potential areas are basically distributed in central China (Shaanxi, Henan, Hubei), so it shows a stable trend of high potential development in central China.

In view of the above phenomena, this study further puts forward four policy recommendations:

1. Reconstruct the cooperation of resource endowment regions. At present, the flow of elements between different regions has shown a stable state, and the accumulation of elements has basically formed a prototype. Therefore, regions with different development potential should form a cooperation mechanism with the surrounding areas according to their own advantageous elements, break through the limitation of their own ' resource curse ', and stimulate the spatial linkage effect for the development of advantageous elements. Relevant regions can consider opening special traffic lines and other ways to open up space and time obstacles between different regions, reduce the circulation cost of resource elements, and provide a path for the flow of advantageous resources. At the same time, we should actively establish cooperation mechanisms with areas with complementary advantages, carry out multi-sectoral integration, set up specialized agencies to be responsible for factor reallocation, and promote the process of efficient resource allocation.
2. According to the production efficiency to promote the reallocation of factors. For high development potential areas, there are only two ways to improve their own development efficiency. One is to improve the development level of the surrounding areas and form an interactive effect, which can be achieved through the introduction of inter-regional cooperation policies. The second is to reconstruct the regional factor allocation structure, optimize production technology, improve the level of technological innovation, so as to improve the development efficiency, which can further promote the development of innovative industries, promote the cultivation and introduction of knowledge-based talents, and break the limitations of traditional production methods by improving the level of technological innovation. For areas with low development potential, investment should be first attracted. The impact of capital on low-potential areas is far greater than other factors. The government can introduce a series of industrial support policies, such as reducing corporate taxes and providing preferential policies for land use. Other areas with development potential should first explore the transformation of energy use structure. The influence of energy factors is more important for these areas. It can further promote the support policies of green industries, deeply apply clean energy to people-benefit projects, and achieve rapid development from green benefits.
3. To further promote the application of low-carbon policy. The input of clean energy has a relatively large role in promoting the development of environmental and economic benefits compared with other factors. In this regard, for each province, based on the requirements of low-carbon policies, we can gradually transition from the use of traditional energy to the use of renewable energy, and actively apply renewable energy to public transportation, green benefit projects and other projects. At the same time, local governments should also effectively supervise and guide the region, attach importance to the positive role of technological innovation in environmental protection, and further create a better development environment for enterprises, constantly stimulate the innovation power of enterprises, and achieve the goal of low carbon. In addition, the government should also give full play to the guiding role of the market while formulating policies. It is necessary to promote enterprises to change their development concepts, encourage enterprises to invest in environmental governance and innovate in green technology, and guide enterprises to actively assume social responsibility, laying the foundation for environmental protection and economic development.
4. Adjust the center of the development potential area at the national level. Although the regions with different development potential show a state of centering on economically developed regions and resource-rich regions, the same model of weakening is presented within these regions. For example, the northeastern region of China is surrounded by development potential around Liaoning, the northwestern region of China is surrounded by development potential around Shaanxi, and the central and western regions are surrounded by development potential around Hubei and Sichuan. Promoting the in-depth development of these core areas at the national level can theoretically effectively drive and radiate the surrounding areas and provide guarantee for the overall development efficiency.
  This study is based on the reconstruction of the traditional directional distance function and the Malmuquist-Luenberger index. Starting from K-means clustering, it innovatively realizes the homogeneous division of inter-regional development efficiency, and uses the center value of the cluster to reflect the change state of the same cluster, which provides a new idea for formulating a relatively accurate research framework. Although this study has expanded the existing research from the basic framework, there are still some deficiencies: (1) In the visualization part, there is a lack of a more accurate gradient process, and only the categories of cluster clusters are used for division. Future research can further explore the efficiency changes in continuous space, which can effectively assist in identifying the connections between different regions. (2) In the identification part of the total factor productivity change index, only the clustering center of the cluster has been used to explore the common characteristics between regions, and there is a lack of research on differentiation. Future research can provide a fundamental basis for the accurate development of the region itself by deeply exploring the differentiated part. (3) The amount of data is relatively small, based on the principle of big data 'related rather than causal '. When the amount of data approaches infinity, a more accurate correlation between potential influencing factors and factor changes can be discovered, which will lay a theoretical and technical foundation for breaking through traditional calculations in the future.

## Data Availability

Most of data can be found in ‘China Energy Statistical Yearbook (https://www.zgtjnj.org/navipage-n3019090603000128.html)’, ’China City Statistical Yearbook ‘(https://www.zgtjnj.org/navibooklist-n3020013291-1.html), and so on. Detailed data presented in this study are available on request from the corresponding author..
